# Modulation of hepatic PPAR expression during *Ft *LVS LPS-induced protection from *Francisella tularensis *LVS infection

**DOI:** 10.1186/1471-2334-10-10

**Published:** 2010-01-18

**Authors:** Saroj K Mohapatra, Leah E Cole, Clive Evans, Bruno W Sobral, Josep Bassaganya-Riera, Raquel Hontecillas, Stefanie N Vogel, Oswald R Crasta

**Affiliations:** 1 Virginia Bioinformatics Institute, Virginia Polytechnic Institute and State University, Blacksburg, VA 24061, USA; 2 Department of Microbiology and Immunology, University of Maryland School of Medicine, Baltimore, MD 21201, USA

## Abstract

**Background:**

It has been shown previously that administration of *Francisella tularensis *(*Ft*) Live Vaccine Strain (LVS) lipopolysaccharide (LPS) protects mice against subsequent challenge with *Ft *LVS and blunts the pro-inflammatory cytokine response.

**Methods:**

To further investigate the molecular mechanisms that underlie *Ft *LVS LPS-mediated protection, we profiled global hepatic gene expression following *Ft *LVS LPS or saline pre-treatment and subsequent *Ft *LVS challenge using Affymetrix arrays.

**Results:**

A large number of genes (> 3,000) were differentially expressed at 48 hours post-infection. The degree of modulation of inflammatory genes by infection was clearly attenuated by pre-treatment with *Ft *LVS LPS in the surviving mice. However, *Ft *LVS LPS alone had a subtle effect on the gene expression profile of the uninfected mice. By employing gene set enrichment analysis, we discovered significant up-regulation of the fatty acid metabolism pathway, which is regulated by peroxisome proliferator activated receptors (PPARs).

**Conclusions:**

We hypothesize that the LPS-induced blunting of pro-inflammatory response in mouse is, in part, mediated by PPARs (α and γ).

## Background

Tularemia is caused by the Gram-negative, non-motile, intracellular coccobacillus, *Francisella tularensis (Ft)*, so named after Tulare county of California where the disease was initially identified [1]. Rodents, along with rabbits and hares, are the chief hosts of the bacterium. It is transmitted to humans mostly by insect bites, handling of animal carcasses, and ingestion or inhalation. Symptoms of tularemia typically appear 3 to 5 days after initial contact with the pathogen and include sudden fever, chills, headaches, diarrhea, muscle aches, joint pain, dry cough and progressive weakness; however, the symptoms and the severity of illness, are highly dependent upon the dose and route of inoculation [2]. The number of cases of tularemia has steadily declined in the United States since 1950; between 1990 and 2000, only 1,368 cases of tularemia were reported to the Centers for Disease Control and Prevention (CDC) with an annual average of approximately 120 cases until 2003 [3]. The CDC has classified *Ft *as a Category A agent due to its low infectious dose, easy dissemination by the aerosol route, and potential to cause severe morbidity and mortality. *Ft *has been used previously as a biological weapon [4]. Furthermore, during the cold war, both the United States and the former Soviet Union stockpiled *Ft *for use as a potential biological weapon [2,5].

An attenuated "Live Vaccine Strain" (LVS), developed in the former Soviet Union by repeated passage of *Ft *subspecies *holarctica *on agar plates and subsequently through mice [6], has been used to vaccinate humans. While vaccination with *Ft *LVS provides significant protection against more highly virulent strains [7-9], the strain has not been licensed for general public use in the United States due to the fact that the molecular basis for the attenuation is presently unknown [10]. Nonetheless, *Ft *LVS is virulent in mice by many routes and causes an infection that resembles human tularemia [11].

Infection of mice with *Ft *results in a marked inflammatory response that has been suggested to be responsible for most of the tissue damage associated with human tularemia [11,12]. Mice challenged i.p. with *Ft *LVS display the highest bacterial burden in the liver when compared to spleen and lung [13]. Interaction of *Ft *with cells of the innate immune system (macrophages, dendritic cells, neutrophils, NK cells) initiates a cascade of cytokine production including IFN-γ, TNF-α leading to production of reactive nitrogen and oxygen species [14]. In the liver, increased bacterial burden is accompanied by increased hepatic mRNA synthesis of the pro-inflammatory genes TNF-α, IFN-γ, KC, MCP-1, and iNOS. A later wave of gene expression is associated with the development of "alternatively activated" macrophages that facilitates increased intracellular replication of *Ft *and, ultimately, death [15].

LPS, an integral structural component of the outer membrane of all Gram-negative bacteria, is often a primary mediator of host inflammatory sequelae induced by Gram-negative bacterial infection. Unlike enterobacterial LPS, previous studies have shown that *Ft *LVS LPS is weakly endotoxic and a poor Toll-like receptor (TLR) 4 agonist [16]. Despite its lack of endotoxic properties, mice pre-treated with *Ft *LVS LPS two days prior to lethal *Ft *LVS challenge are protected and display decreased bacterial burden as well as a reduced inflammatory response [13]. This previous work assessed changes in gene expression using real-time PCR and, therefore, a relatively small subset of inflammatory genes was analyzed. To gain insights into the mechanism(s) of *Ft *LPS-mediated protection of mice from death caused by *Ft *LVS, we profiled hepatic transcriptome (using Affymetrix expression arrays) of *Ft*-infected mice with or without LPS-pre-treatment.

## Methods

### Sample preparation

Female C57BL/6J mice were obtained from Jackson Labs in Bar Harbor, ME. 48 hours prior to *Ft *LVS challenge, mice were injected i.p. with either 100 ng of *Ft *LVS LPS or an equivalent volume of saline. On the day of challenge, 3 saline- and 3 *Ft *LVS LPS-pre-treated animals were sacrificed (uninfected controls), while all remaining mice were challenged i.p. with ~4-5 × 10^5 ^*Ft *LVS. *Ft *LVS-challenged mice were sacrificed (in groups of 3) at 24 and 48 hours post-infection. This entire experiment was performed 3 times. Within each individual experiment, equal amounts of extracted RNA from biological replicate samples were pooled. Pooled RNA from separate experiments was used as biological replicates. All experimental procedures were approved by the Institutional Animal Care and Use Committee and met or exceeded requirements of the Public Health Service/National Institutes of Health and the Animal Welfare Act.

### RNA extraction

Whole liver was collected from mice and preserved in RNAlater^® ^(Applied Biosystems/Ambion Foster City, CA). After homogenization, total RNA was extracted and purified using the RNAeasy system according to manufacturer's instructions (Qiagen Valencia, CA). The QIAGEN RNase-free DNase supplement kit was used to ensure that the RNA was free from DNA contamination. All RNA samples were checked for both quality and quantity as described previously [17,18].

### Real Time PCR

Real-time PCR was performed in a Sequence Detector System (ABI Prism 7900 Sequence Detection System and software; Applied Biosystems, Foster City, CA) as described previously [19]. Levels of mRNA for specific murine genes were reported as relative gene expression over background levels detected in control samples. Primers were designed using the Primer Express™ Program (Applied Biosystems, Foster City, CA) in conjunction with GenBank with the following sequences.

B-cell leukemia/lymphoma 6 forward: GTCAGAGTATTCGGATTCTAGCTGTG 

B-cell leukemia/lymphoma 6 reverse: GCAGCGTGTGCCTCTTGAG

Traf 2 binding protein (TIFA) forward: GGCCACTGGAAGACTCTCAGG

Traf 2 binding protein (TIFA) reverse: GGATGGTAAATGGTCATCTGGAG

cytochrome P450, family 2, subfamily b, polypeptide 10 (Cyp2b10) forward: CAGACACCATAAGGGAGGCTCT

cytochrome P450, family 2, subfamily b, polypeptide 10 (Cyp2b10) reverse: GATCACACCATATTCCTTGAAGGTT

Heat shock protein 1B (Hspa1b) forward: GCACGGCGTGTGAGAGG

Heat shock protein 1B (Hspa1b) reverse: TGATGGATGTGTAGAAGTCGATGC.

### GeneChip hybridization

RNA was processed and labelled according to the standard target labelling protocols and the samples were hybridized, stained, and scanned per standard Affymetrix protocols at VBI core laboratory on Mouse 430 2.0 expression arrays (Affymetrix Inc., Santa Clara, CA). This platform consists of 45,101 probe sets representing 21,309 genes of the mouse genome.

### Microarray data analysis

Data input and subsequent steps were performed using the Bioconductor package "affy" [20] in R statistical environment - Version 2.8.1 [21]. Raw microarray data obtained from CEL files were pre-processed by the gcRMA algorithm (GC Robust Multiarray Average) [22] that performs the three steps: (i) adjustment of the gene expression signal against the background caused by optical noise and non-specific binding, (ii) robust multi-array normalization [23], and (iii) summarization of the probes belonging to each probe set.

The probe sets were further selected in an unbiased manner by removing those associated with a very low degree of variability (inter-quartile range less than 0.5) across all the samples. This reduced the number of probe sets from 45,101 to 19,535. Statistical analysis was performed using the software package "limma" [24] by applying a linear model to the expression measurement (log intensities) for each gene. For each comparison of interest, the genes were assigned p-values after controlling for false discovery rate [25]. For assessment of differential expression, the empirical Bayes method was applied [26]. A cut-off of p < 0.05 and > = 2-fold change was used as the criterion for whether or not a gene was significantly modulated by a treatment. All the significantly modulated genes in any of the pair wise comparison of interest are listed in Additional file 1. The microarray data (both raw and normalized) have been submitted at the Gene Expression Omnibus (GEO, http://www.ncbi.nlm.nih.gov/geo/, Data set: GSE16207).

### Gene set enrichment analysis (GSEA)

All pathways listed at KEGG [27-29] were selected for analysis. The degree of differential regulation of a pathway was derived from the collective differential regulation of the member genes in that pathway, as described below. Each pathway was assigned a score (Z_K_) by combining individual t-statistic of the genes in that pathway, using the following formula [30]:

where the *t*-statistic is a measure of the individual differential expression for each gene in the pathway. While each individual t-statistic represents the gene-level difference between two groups (treated versus control), the pathway score represents the pathway-level difference between the two groups. Calculation of the pathway score was performed using the bioconductor package GSEABase [31]. GSEA has earlier been shown to detect differential regulation of pathways when gene-level changes are very small or undetectable [32,33] and hence was considered appropriate for the present context. The pathway observed to be most significantly modulated was confirmed by permutation testing with the gseattperm function in the package Category [34]. The pathway scores are listed in Additional file 2.

### Comparison of differential gene expression due to LPS treatment

In view of the regulatory role of peroxisome proliferator-activated receptors (PPARs) in fatty acid metabolism, the gene expression of three PPAR isoforms (PPARα, PPARγ, PPARβ/δ) were subjected to further analysis. Our goal was to detect if post-infection gene expression of PPAR was consistently elevated by LPS pre-treatment. Hence a t-test (paired by time-point) between two conditions (LPS and No LPS) was performed for each isoform, as described below. For each time point (uninfected, 24 hr PI and 48 hr PI), differences between the two treatments were calculated as d_0_, d_1 _and d_2 _respectively, using the formulas below.

Each of the values d0, d1, and d2 refers to the difference of the average gene expression of 3 samples. The average and variation of these numbers were compared against a normal distribution and tested if the mean was significantly different from zero. For PPARα and γ (but not β/δ), the effect was found to be statistically significant (p < 0.05).

### Comparison of differential gene expressions due to LPS treatment and infection on fatty acid metabolism

A similar method as described above was applied to assess the effect of infection and LPS treatment on fatty acid metabolism. In this case, our intention was to compare the average direction of change in the expression of a total of 24 genes involved in fatty acid metabolism after challenging the mice with LPS alone (48 hours) or infection alone (24 hours). The time point of 24 hours post-infection was chosen to detect the early changes post-infection. For each gene, differences of gene expression due to LPS (Dp) and due to infection (Di) were calculated as follows:

For each gene, it refers to the difference of the gene expression values averaged over 3 samples. The average and variation of these numbers (Dp and Di) were compared against a normal distribution and tested if the mean was significantly different from zero. For each case (LPS or infection), the average differences of Dp and Di were tested against normal distribution found to be significantly different from zero (p < 0.01).

## Results

### Alteration in gene expression after infection

We discovered 3,515 genes to be differentially expressed after 48 hours of infection. These include Bcl proteins 3, 6, and 10; heat shock proteins 1, 1A, 1B, and 90; genes induced by interferons; interleukin 1β; and LPS binding protein and tumor necrosis factor. For easier data visualization, a shorter list of 79 of these genes (with >40-fold change) is presented in the heat map in Additional file 3 A. Genes with modified expression fell into two categories based on whether they were up or down-regulated by infection. The up-regulated genes include interferon γ (IFN-γ) as well as chemokine (C-X-C motif) ligands IP-10 (Cxcl10), KC (Cxcl1), and MCP-1 (Ccl2). This is consistent with our previously published results on up-regulation of pro-inflammatory cytokines by mouse liver upon *Ft *LVS infection [13]. The genes that were down-regulated at 48 hours post-infection include carbonic anhydrase 3 and guanidinoacetate methyltransferase, both of which are known to be down-regulated in response to liver injury [35,36].

The time course for gene expression for up- and down-regulated genes was examined separately. Box plots were drawn for each group of genes across the three time points: uninfected, 24 hours, and 48 hours post-infection (Additional file 3B, C). For each post-infection time point, saline- and LPS-pre-treated mice were juxtaposed. Two trends are visible in these box plots. First, with time, infection progressively alters the level of gene expression (red-colored boxes). This is true for both up- and down-regulated genes. Secondly, LPS-pre-treatment (green boxes) opposes the transcriptional changes caused by *Ft *LVS infection at both 24 hours and 48 hours post-infection, thus keeping the gene expression level less deviated from uninfected state. Effect of LPS-pre-treatment was found to be statistically significant (p < 0.01) for both up- and down-regulated genes.

We discovered a total of 34 genes to be differentially expressed compared to uninfected control. These included chemokine ligand IP-10 and genes controlled by pro-inflammatory cytokines TNF-α, IL-1β, IFN-γ; namely, interferon regulatory factor 1, Cd274 antigen, metallothionein 2, proteasome subunit (Psmb9), and tumor necrosis factor Tnfsf10 were progressively up-regulated after infection. Similar to the trend seen for the other group, up-regulation of these genes at 24 hours post-infection was lessened by LPS-pre-treatment.

In addition to the effects on the specific genes, LPS-pre-treatment had a substantial influence on the number of genes differentially expressed due to infection. A total of 3,515 genes were found to be differentially expressed in mice without LPS-pre-treatment, but only 1,494 genes with LPS-pre-treatment after 48 hours post-infection as compared to the uninfected controls (0 hr post-infection). For more than 99% of the latter list of genes (1,483 out of 1,494) the N-fold was in the same direction as that in mice without LPS-pre-treatment. Furthermore, for 1,100 of these 1,483 genes, the magnitude of change was smaller in the LPS pre-treated mice, suggesting LPS had a general blunting effect on infection-induced gene expression profile.

### Validation of select genes

**Figure 1:**
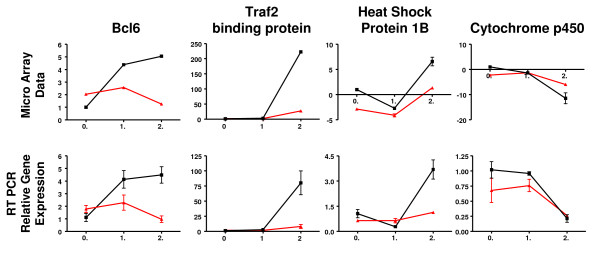
**Real-time PCR of 4 genes**. Measurement of relative gene expression in infected mice (with respect to uninfected mice without LPS-pre-treatment) of four different genes by two different methods. The top row corresponds to microarray while the bottom row corresponds to quantitative real-time PCR. In each plot, the red line corresponds to the group of mice with LPS-pre-treatment, the black line, without LPS. Horizontal axis corresponds to the days after infection, vertical axis to relative gene expression. Gene expression trends are similar between the two methods of measurement.

**Figure 2:**
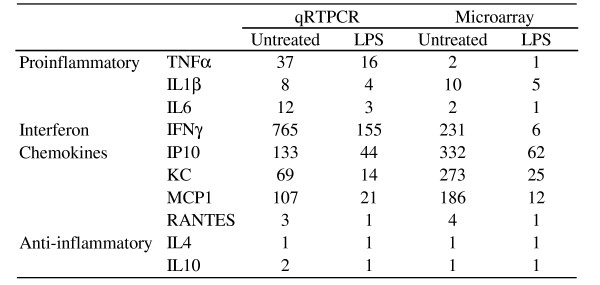
**Agreement with previously published result**. Agreement of microarray data with gene expression changes measured by quantitative real-time PCR (qRTPCR) [13]. In both studies the mice were pre-treated with either 100 ng of *Ft *LVS LPS or equivalent volume of saline (Untreated) 48 hours before infection and sacrificed at 48 hours post-infection. Each number represents the fold-increase in the mRNA levels after infection. While there was rise in the pro-inflammatory cytokine levels post-infection, LPS caused a reduction in expression of these genes (comparable between qRTPCR and microarray; Spearman correlation at p < 0.001).

Validation of the data from a selected number of genes was carried out by quantitative real-time RT-PCR (qRTPCR). Relative gene expression of four genes (Bcl6, Traf2 binding protein, heat shock protein 1B, and Cytochrome P450) was measured by qRTPCR. These genes were found to have similar trends as seen on the microarrays (Fig. 1). Another set of genes (TNF-α, IL-1β, IL-6, IFN-γ, IP-10, KC, MCP-1, RANTES) reported earlier to be up-regulated [13] were also examined. Both microarray and qRTPCR measurements for these genes were consistent with respect to the direction of change at a statistically significant level (Fig. 2).

### LPS-induced changes in pathways

**Figure 3:**
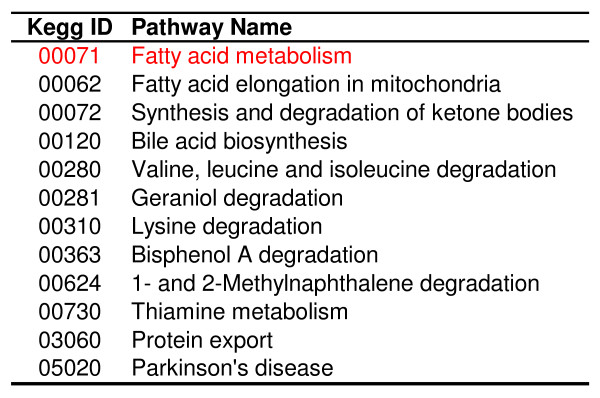
**List of significant pathways**. List of pathways found to be significantly up-regulated by permutation testing of the KEGG pathways. The pathway Fatty acid metabolism (KEGG ID: 00071) was found to have the highest pathway score (Additional file 2). Many genes participating in fatty acid metabolism are regulated by PPARs.

While LPS-pre-treatment blunted up-regulation of genes at 24 and 48 hours post-infection, no significant changes in gene expression could be demonstrated in the livers of mice that received LPS and were sacrificed prior to *Ft *challenge (i.e., 48 hours after LPS injection). This finding suggests that the earliest transcriptional changes were subtle or that the initial modulation of the host response to *Ft *occurred in an extra-hepatic location. In order to enhance the discriminatory power of the small changes at the level of individual gene expression, we employed a GSEA analytic strategy that relies on coordinated expression of functionally related genes. We first compared saline- and LPS-treated mice at time point zero, which is equivalent to measuring the effect on global gene expression profile after 48 hours of LPS treatment. Pathway scores were calculated between the two conditions. The most up-regulated pathway "Fatty acid metabolism" (KEGG ID: 00071) was found to be statistically significant from permutation testing (Fig. 3). Many of the genes in this pathway are transcriptionally regulated by PPARs.

PPARs belong to the superfamily of nuclear hormone receptors with three known isoforms, α,β or δ, and γ, that differ in their tissue distribution and functional activity [37]. While PPARβ/δ is ubiquitous, PPARγ is expressed in adipocytes, T- and B-cells, monocytes/macrophages, dendritic cells, and epithelial cells [38,39].

**Figure 4:**
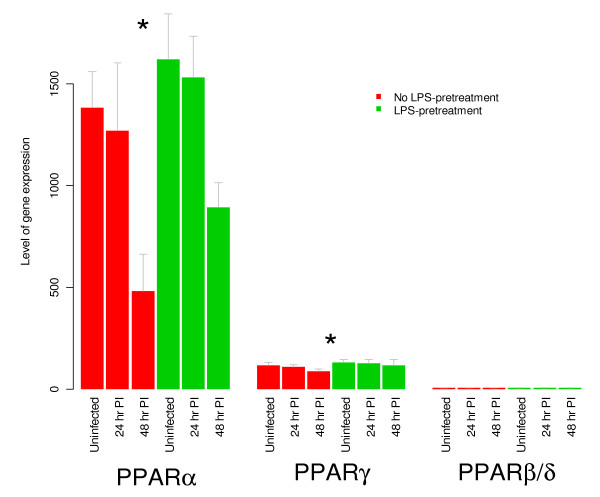
**Expression of PPAR genes in mouse liver**. Hepatic gene expression levels of three different peroxisome proliferator-activated receptor genes. Gene expression level was calculated from normalized intensities for six groups of samples. Error bars correspond to standard deviation among the three biological replicates. PPARα is the most highly expressed of the three genes (followed by PPARγ). Furthermore, effect of LPS treatment was assessed for each gene by using a paired t-test (please see details in subsection "Comparison of specific gene expression" under Methods section). The asterisk indicates that LPS treatment causes significant up-regulation (p < 0.05) of PPARα and PPARγ, but not PPARβ/δ.

**Figure 5:**
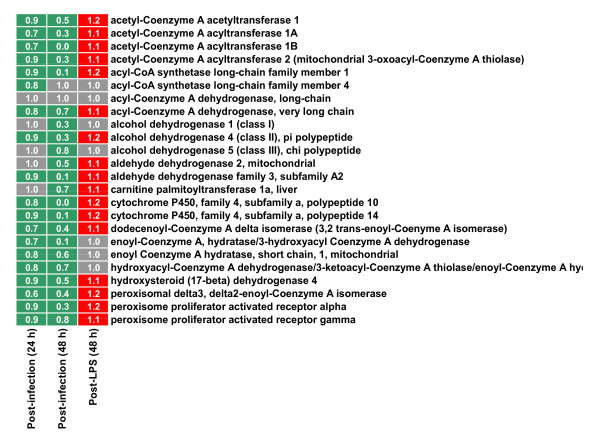
**Modulation of genes belonging to fatty acid metabolism by LPS and infection**. Heat map showing the effect of infection and LPS on transcription of genes participating in fatty acid metabolism, including the genes PPARα and γ. The three columns correspond to the effect of *Ft *LVS infection alone (24 and 48 hour post-infection) and LPS alone (48 hour post-LPS). The treated group (post-infection or post-LPS) was compared with the untreated control for calculation of fold change (numbers inside the cells). The green color suggests down-regulation (fold change < 1); red color up-regulation (fold change > 1). Genes regulated by PPARα and γ belong to multiple pathways including cellular fatty acid uptake, intracellular fatty acid transport, ketogenesis, lipoprotein metabolism, microsomal fatty acid ω-oxidation, mitochondrial fatty acid β-oxidation, mitochondrial fatty acid uptake, peroxisomal fatty acid β-oxidation, and peroxisomal fatty acid uptake [41]. Most genes (participating in fatty acid metabolism) are up-regulated after LPS treatment (red color of the cells in the right column) but are down-regulated after infection (green color in the left column). Both post-infection and post-LPS changes in expression of this set of genes were observed to be statistically significant as detected by paired t-test between treated and control groups (p < 0.01; please see details in subsection "Comparison of specific gene expression" under Methods section).

However, it is PPARα that is reported to be highly expressed in liver cells [40]. We found that, under all conditions and at all time-points, the level of PPARα gene expression is highest in the liver followed by PPARγ (Fig. 4). PPARα forms a heterodimer with retinoid X receptor (RXRα) that binds to DNA on PPAR response elements and controls the expression of a number of genes participating in metabolism. The gene-products regulated by PPARα participate in a number of metabolic processes including cellular fatty acid uptake, intracellular fatty acid transport, ketogenesis, lipoprotein metabolism, microsomal fatty acid ω-oxidation, mitochondrial fatty acid β-oxidation, mitochondrial fatty acid uptake, peroxisomal fatty acid β-oxidation, and peroxisomal fatty acid uptake [41]. As shown in the right column of Fig. 5, PPARα, γ, and most of the genes participating in fatty acid metabolism are expressed at a higher level (red) after LPS treatment (fold change > 1) as compared to the uninfected and untreated (No LPS) control. On the contrary, most of the same genes expressed at a lower level (green) after infection (fold change < 1) as compared to the uninfected and untreated (No LPS) control. The averages of these fold changes across all the genes in the fatty acid metabolism was significantly (p < 0.01) higher due to LPS treatment and lower due to infection.

## Discussion

*Ft *LVS infection of mice is associated with profound changes in the levels of pro-inflammatory cytokines whose expression is decreased by pre-treatment with 100 ng of *Ft *LVS LPS. After 48 hours post-infection, LPS-treated mice exhibit significantly reduced hepatic gene expression [13] (Fig. 2), circulating cytokine levels [13] and increased survival [13]. We previously postulated that pre-treating the mice with LPS accomplishes the following results: (i) dampened bacterial growth, (ii) suppressed pro-inflammatory response, and (iii) induction of a late Ag-specific protective adaptive immune response [13]. It was recently shown that the latter is due to the induction of *Ft *LVS LPS-specific antibodies by a subset of B1a cells [42]. Upon global profiling of hepatic gene expression, we discovered a number of genes that were differentially expressed, especially at 48 hours post-infection, a time at which many of these changes were counteracted by prior LPS treatment. The effect of LPS treatment was less evident at 24 hours post-infection or at day 0 (without infection). Since it is clear that LPS protects the mice from death by *Ft *LVS, we made the reasonable assumption that the protective molecular changes were already in motion 48 hours after LPS treatment (day 0) and we sought to discover the gene expression signature of those LPS-induced changes. Using "Gene set enrichment analysis" that explores coordinated changes in functionally related genes, we discovered that the KEGG pathway "Fatty acid metabolism" was up-regulated at 48 hours post-infection. Further investigation pointed to simultaneous up-regulation of PPARα and γ by LPS-pre-treatment (Fig. 5).

Many of the genes in the "Fatty acid metabolism" pathway are transcriptionally controlled by peroxisome proliferator-activated receptors (PPARs). PPARs (α, β/δ and γ) are master regulators of energy homeostasis. They are nuclear receptors that upon activation by specific ligands bind to specific response elements near the promoter of their target genes. In this way, PPARs sense the lipid concentration and composition in the cellular environment [43,44]. In fact, PPARs are the receptors for endogenous lipid molecules such as prostaglandins or hydroxy-containing PUFA such as 12/15-hydroxyeicosatetraenoic (HETE), 13-hydroxyoctadecadienoic (HODE), and dietary compounds such as conjugated linoleic acid. PPARα and γ were originally identified as the molecular target for the fibrate class of lipid-lowering drugs or the thiazolidinedione (TZD) class of antidiabetic drugs, respectively. Our results show that the down-regulation of the proinflammatory cytokine and chemokine response induced by LPS pre-treatment is paralleled by increased expression of PPARα and γ. In the liver, PPARα is mainly expressed by hepatocytes, where it regulates oxidation of free fatty acids. Its ligands include unsaturated fatty acids and eicosanoids derived from arachidonic and linoleic acids. We show that LPS-pre-treatment resulted in up-regulation of the enzymes involved in fatty acid oxidation (Fig. 5), which suggests a higher degree of PPARα activation by endogenous ligands.

Both PPARα and γ are expressed in Kupffer cells, a specialized subset of macrophages that reside in the hepatic sinusoids [45]. Other immune cells present in the liver such as dendritic cells or T cells also express PPARγ[46]. In addition to its role in lipid metabolism, PPARα and γ have long been recognized for their influence on inflammatory pathways [47] mostly by repression of pro-inflammatory gene expression. More recently, it has been shown that liver PPARα controls acute phase response (APR) via a liver-specific attenuation of pro-inflammatory cytokine gene expression [48]. In this model, PPARα modulates both TNF-α/IL-1 and IL-6 signaling through a direct action on the pathway or via the down-regulation of IL-1-mediated stimulation of IL-6 expression. This model is consistent with our finding of up-regulated PPARα-responsive genes and reduced levels of pro-inflammatory cytokine gene expression and protein levels. Interestingly, while purified *Ft *LPS causes an up-regulation of PPARs and their responsive genes, infection with the whole bacterium causes down-regulation of the same genes (Fig. 5). These findings are in line with a recent report demonstrating that *E. coli *LPS up-regulated PPARγ expression in the immune system of pigs by inducing the generation of endogenous PPARγ agonists such as 15d PGJ (2) [49]. Thus, while enterobacterial and *Ft *LPS differ in molecular targets (i.e., TLR4 versus TLR2, respectively) and their ability to induce inflammatory cytokines, both types of LPS may induce the generation of endogenous PPAR agonists as a mechanism of down-regulating inflammation.

PPARγ has been recently shown to be a key molecular switch in the induction of alternatively activated macrophages [50]. Moreover, it has been shown that infection of macrophages with *Ft *LVS induced alternative activation with up-regulation of the signature genes arginase I, FIZZI, and macrophage mannose receptor [15]. Interestingly, the cytokines IL-4 and IL-13 drive differentiation into alternatively activated macrophages through IL-4Rα and STAT6 signaling, [51] and in addition, IL-4 treatment of macrophages up-regulates PPARγ and PPARγ responsive genes [52]. While alternatively activated macrophages display an anti-inflammatory phenotype, which might dampen *Ft *LVS-induced inflammation, they are unable to kill intracellular pathogens. In this regard, *Ft *LVS induced alternatively activated macrophages through production of IL-4 and IL-13, which resulted in enhanced intracellular bacterial survival and replication. On the other hand, the inability of macrophages to become alternatively activated in IL-4Rα or STAT6-deficient mice leads to a more prolonged IL-12 response when compared to WT littermates [15]. Thus, the role of PPARγ in the immunopathogenesis of tularemia should be interpreted with caution as it might have both beneficial and detrimental effects in the outcome of the disease. The induction of the PPAR-controlled pathway in our data is consistent with transcriptional profiling results of *F. tularensis *infection of human peripheral blood mononuclear cells (PBMC) [53].

## Conclusions

In summary, we have examined the gene expression of the mouse liver before and after infection with *Ft *LVS, with or without LPS-pre-treatment, and demonstrated that, in accordance with previous results from our laboratory, LPS causes significant attenuation of gene-expression changes after infection with *Ft *LVS. Additionally, we demonstrate for the first time that *Ft *LPS causes subtle changes in hepatic gene expression after 48 hours of treatment in uninfected mice. By using gene set enrichment analysis (GSEA) we showed that many genes involved in fatty acid metabolism are up-regulated in a coordinated manner along with up-regulation of the nuclear factor PPARα (and to a lesser extent PPARγ). We suggest that LPS-induced attenuation of pro-inflammatory response to *Ft *LVS is partly mediated through hepatic PPARα and γ, possibly through the induction of alternatively activated macrophages. These findings are in line with numerous reports on the anti-inflammatory efficacy of PPARs. Future studies aimed at examining the effect of *Ft *LVS or type A strains on the hepatic and pulmonary pathology following cell-specific deletion of PPARα or γ from immune cells are needed to further dissect the role of PPARs in the pathogenesis and prevention of tularemia.

## Competing interests

The authors declare that they have no competing interests.

## Authors' contributions

SV, LC, OC designed the experiments; LC, SV did the mouse experiments; CE did the microarray processing of samples; SM, OC performed statistical analysis of microarray data; SM, LC, JB, RH, SV, OC interpreted the results; SM, LC, JB, RH, SV, OC wrote the paper; BS provided helpful comments. All authors reviewed and approved of the manuscript.

## Pre-publication history

The pre-publication history for this paper can be accessed here:

http://www.biomedcentral.com/1471-2334/10/10/prepub

## Supplementary Material

Additional file 1 **Genes showing significant modulation after 48 hours of infection**. All the significantly modulated genes in any of the pair wise comparison of interest (contrasts) are listed. There are seven contrasts: LPS_d0_Vs_Sal_d0, LPS_d1_Vs_Sal_d1 LPS_d2_Vs_Sal_d2 Sal_d1_Vs_Sal_d0, Sal_d2_Vs_Sal_d0, LPS_d1_Vs_LPS_d0 LPS_d2_Vs_LPS_d0. For each, fold change and p-values are provided. Additionally, selection columns include 1 for significant differential expression and 0 for no significant differential expression. Click here for file

Additional file 2 **Pathway score for the KEGG pathways**. Pathway score was calculated from the gene-level fold-change in expression induced after 48 hours of LPS treatment. Fatty acid metabolism appears at the top of the list. Click here for file

Additional file 3 **LPS lessens the impact of *Ft *LVS infection on mouse liver**. Gene expression changes induced by *Ft *LVS infection and protection by LPS pre-treatment. (A) Heat map showing gene expression before (Uninfected) and after infection (48 hours PI). Higher degree of gene expression is displayed as a darker cell. This is a set of selected genes showing maximal difference (absolute fold change of 40 or higher) between the two groups of mice. The genes are either up- or down-regulated by infection. Some of the genes associated with inflammation or liver injury have been outlined: up-regulated in red rectangle; down-regulated in green rectangle. The highlighted genes have already been reported to be altered in response to *Ft *infection or liver injury. (B) Distribution of expression signal of genes up-regulated by infection. Each box plot corresponds to distribution of the up-regulated genes before or after infection (24 hours, 48 hours PI). The red color corresponds to mice without LPS pre-treatment. The green color corresponds to mice with LPS pre-treatment. (C) Same as B, but for down-regulated genes. In both B and C, infection causes progressive alteration of transcription for these genes and LPS pre-treatment opposes this trend. Click here for file
