# Supplementary material for: Modulation of hepatic PPAR expression during Ft LVS LPS-induced protection from Francisella tularensis LVS infection
Source: BMC Infect Dis. 2010 Jan 18;10:10. doi: 10.1186/1471-2334-10-10 (PMC2826305; doi:10.1186/1471-2334-10-10)
Supplement: Additional file 3 — LPS lessens the impact of Ft LVS infection on mouse liver. Gene expression changes induced by Ft LVS infection and protection by LPS pre-treatment. (A) Heat map showing gene expression before (Uninfected) and after infection (48 hours PI). Higher degree of gene expression is displayed as a darker cell. This is a set of selected genes showing maximal difference (absolute fold change of 40 or higher) between the two groups of mice. The genes are either up- or down-regulated by infection. Some of the genes associated with inflammation or liver injury have been outlined: up-regulated in red rectangle; down-regulated in green rectangle. The highlighted genes have already been reported to be altered in response to Ft infection or liver injury. (B) Distribution of expression signal of genes up-regulated by infection. Each box plot corresponds to distribution of the up-regulated genes before or after infection (24 hours, 48 hours PI). The red color corresponds to mice without LPS pre-treatment. The green color corresponds to mice with LPS pre-treatment. (C) Same as B, but for down-regulated genes. In both B and C, infection causes progressive alteration of transcription for these genes and LPS pre-treatment opposes this trend. [file 1471-2334-10-10-S3.PDF]

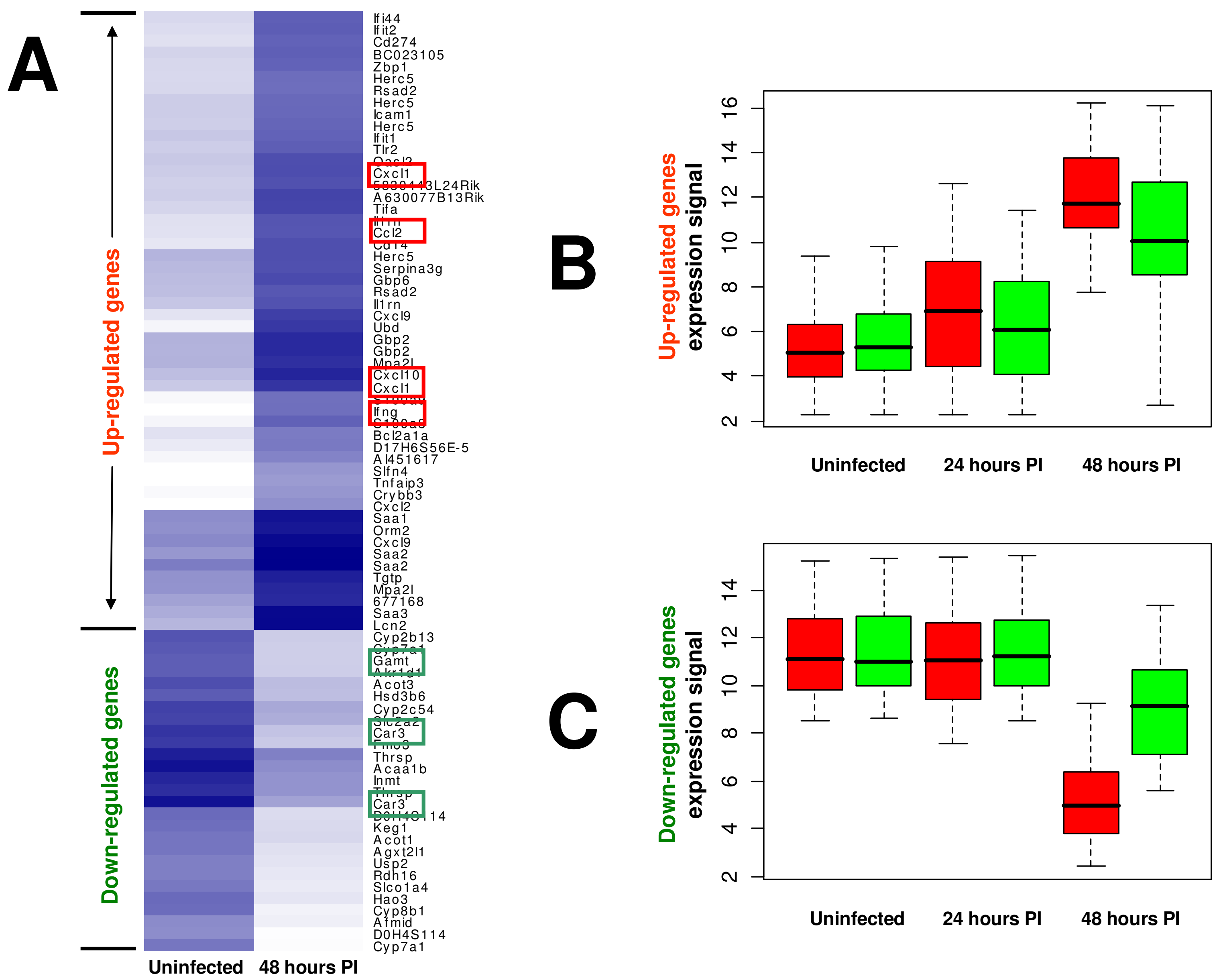

A
B
C
Up-regulated genes
Down-regulated genes
Uninfected
48 hours PI
Ifi44
Ifit2
Cd274
BC023105
Zbp1
Herc5
Rsad2
Herc5
Icam1
Herc5
Ifit1
Tlr2
Cxcl1
A630077B13Rik
Tifa
Ccl2
Herc5
Serpina3g
Gbp6
Rsad2
Il1rn
Cxcl9
Ubd
Gbp2
Gbp2
Cxcl10
Cxcl1
Ifng
Bcl2a1a
D17H6S56E-5
AI451617
Slfn4
Tnfaip3
Crybb3
Cxcl2
Saa1
Orm2
Cxcl9
Saa2
Saa2
Tgtp
Mpa2l
677168
Saa3
Lcn2
Cyp2b13
Gamt
Acot3
Hsd3b6
Cyp2c54
Car3
Thrsp
Acaa1b
Inmt
Car3
Keg1
Acot1
Agxt2l1
Usp2
Rdh16
Slco1a4
Hao3
Cyp8b1
Afmid
D0H4S114
Cyp7a1
Up-regulated genes expression signal
Down-regulated genes expression signal
2
4
6
8
10
12
14
16
Uninfected
24 hours PI
48 hours PI
